# Reasons for Childhood Vaccine Refusal: A Cross-Sectional Study in South-Eastern Türkiye

**DOI:** 10.3390/children13091247

**Published:** 2026-09-15

**Authors:** Murat Solmaz, Selami Yunus Ertek, Ali Nazım Güzelbağ, Özhan Orhan

**Affiliations:** 1 Department of Pediatrics, Republic of Turkey Ministry of Health, Batman 72060, Turkey; 2 Department of Family Medicine, Republic of Turkey Ministry of Health, Batman 72060, Turkey; 3 Department of Pediatric Cardiology, Mardin Training and Research Hospital, Mardin 47100, Turkey; 4 Department of Pediatrics, Mardin Artuklu University, Mardin 47100, Turkey

**Keywords:** vaccine refusal, vaccine hesitancy, childhood immunisation, parental attitudes, misinformation, Türkiye

## Abstract

**Highlights:**

**What are the main findings?**

- Among 4118 documented childhood vaccine refusals in a south-eastern Turkish province, parents most often cited media and community reports questioning vaccine safety (71.3%), and reported a mean of 3.6 reasons each rather than a single concern.
- The reasons parents gave differed by socioeconomic position: doubts about vaccine safety were more common in lower-income and less-educated families, whereas the belief that vaccination is unnecessary was more common in higher-income families.
- Concern about side effects was common at every educational level, including among university graduates, indicating that refusal was not limited to less-educated parents.

**What are the implications of the main findings?**

- Because refusal rested on a cluster of mutually reinforcing beliefs, communication efforts that correct a single misconception are unlikely to be sufficient on their own.
- Messages should be tailored to each group: building trust and countering misinformation where confidence is low, and restoring awareness of disease risk where families see vaccination as unnecessary.
- Routine primary care visits remain the key opportunity to address parental concerns directly, supporting investment in the communication skills of family physicians and nurses.

**Abstract:**

**Background/Objectives:** Vaccine hesitancy has intensified worldwide, and measles and pertussis have resurged across the WHO European Region. In Türkiye, a regulation introduced in May 2024 made documentation of every parental refusal mandatory, rendering 2025 the first calendar year for which provincial refusal data can be considered systematically ascertained. **Methods:** We conducted a retrospective, registry-based cross-sectional study of all vaccine refusal notifications submitted to the Batman Provincial Health Directorate between 1 January and 31 December 2025, covering all six districts of the province. Sociodemographic data were extracted from the notification database, and reasons for refusal were obtained using a structured questionnaire administered by primary healthcare personnel. Parents could endorse more than one reason. **Results:** A total of 4118 notifications were analysed. Mothers completed 62.4% of notifications; 44.2% of parents had primary education only and 58.3% belonged to low-income households. The diphtheria–tetanus–acellular pertussis–inactivated poliovirus–Haemophilus influenzae type b (DTaP-IPV-Hib) combination vaccine (28.4%) and measles–mumps–rubella (MMR) vaccine (22.1%) were the most frequently refused vaccines. Exposure to media or community reports was the most frequently endorsed reason (71.3%), followed by concern about adverse effects (68.9%) and doubts about vaccine safety (65.4%). Parents endorsed a mean of 3.6 reasons each. Doubts about safety predominated in lower socioeconomic strata, whereas the perception that vaccination is unnecessary predominated in higher strata. **Conclusions:** Refusal in this setting rests on a cluster of mutually reinforcing beliefs rather than a single misconception, and the concerns reported differ systematically by socioeconomic position. Differentiated rather than uniform communication strategies are required.

## 1. Introduction

Immunisation ranks among the most effective and cost-efficient interventions available in preventive medicine. Modelling of the first five decades of the Expanded Programme on Immunization [1] indicates that childhood vaccination has averted an extraordinary burden of premature mortality worldwide, with the largest single contribution attributable to measles-containing vaccines. Notwithstanding this evidence base, the delay in acceptance or the outright refusal of vaccines despite the availability of immunisation services—defined by the World Health Organization (WHO) Strategic Advisory Group of Experts and elaborated by MacDonald et al. [2] as vaccine hesitancy—has emerged as a persistent obstacle to sustaining coverage. The WHO designated vaccine hesitancy as one of the ten leading threats to global health in 2019, and the phenomenon has intensified in the aftermath of the COVID-19 pandemic [3].

The consequences of this trend are now measurable rather than anticipated. According to the WHO–UNICEF Estimates of National Immunization Coverage released in July 2026 [4], 90% of infants worldwide received at least one dose of diphtheria–tetanus–pertussis-containing vaccine during 2025 and 85% completed the three-dose series, yet an estimated 13.5 million children received no vaccine whatsoever in their first year of life.

Regional data illustrate the same pattern with greater granularity. Within the WHO European Region, 53 countries reported 33,998 measles cases in 2025. Although this represented a decline of almost 75% relative to the 127,412 cases recorded in 2024, the figure still exceeded that of most years since 2000. More than 200,000 people in the Region contracted measles across the preceding three years [5]. In January 2026, six countries lost their measles elimination status, while the number of countries with re-established or ongoing endemic transmission rose from 12 to 19 [5,6]. In the United States, 2245 measles cases were recorded across 44 jurisdictions during 2025—the highest annual total since 1991—including three deaths, all in unvaccinated individuals [7], and Canada lost its measles elimination status in November 2025. A parallel resurgence of pertussis has been documented across multiple regions. Within the EU/EEA, nearly 60,000 cases were reported between January 2023 and March 2024, approximately a ten-fold increase relative to 2021 and 2022 [8].

These epidemiological signals are underpinned by a measurable erosion of public confidence. In a large-scale temporal modelling study, de Figueiredo et al. [9] demonstrated that perceived vaccine safety declined in a majority of surveyed countries, with subsequent surveillance indicating further deterioration during and after the pandemic. At the level of individual decision-making, the systematic review and meta-analysis of Abenova et al. [10] estimated that approximately one in five parents of children aged 0–6 years exhibits some degree of hesitancy towards routine childhood vaccination.

The determinants of hesitancy are multifactorial and strongly context-dependent, conventionally framed in terms of confidence in vaccines and providers, complacency arising from low perceived disease risk, and convenience of access [2]. In their systematic review of the global literature, Larson et al. [11] identified parental educational attainment, household income, religious conviction, trust in health institutions and fear of adverse events as recurrent contributors, a synthesis broadly corroborated by Yaqub et al. and Dubé et al. [12,13]. Exposure to misinformation has assumed particular prominence: Puri et al. documented a strong association between social media use and vaccine hesitancy [14], while healthcare providers remain among the strongest modifiable influences on parental vaccination decisions [15]. Socioeconomic gradients have been demonstrated in nationwide European surveys by Bocquier et al. [16], with institutional trust operating as a mediating variable.

Türkiye occupies a distinctive position within this landscape. Vaccines included in the national childhood immunisation schedule are supplied free of charge to all children, including migrant populations, through an accessible performance-based family medicine system underpinned by an integrated national health information infrastructure; vaccination is nonetheless not legally compulsory [17]. Coverage gains have been considerable: Eryurt and Yalçin documented a substantial fall in the prevalence of zero-dose children between 1990 and 2018 [18], although pronounced regional inequities persisted throughout. Paradoxically, refusal rose over the same interval from isolated events to more than 23,000 families by 2018, a trend linked by Gür [17] to intensified public debate following a 2015 judicial decision concerning parental consent. National surveillance encompassing all 80 provinces, reported by Yalçin et al., documented 8977 refusal cases in 2016, corresponding to 3.5 per 1000, rising to 14,779 cases and 5.9 per 1000 in 2017 [19]. Community-based studies conducted over the same period, including those of Kurt et al. and Özceylan et al., consistently identified considerably higher underlying hesitancy, indicating a systematic discrepancy between administrative and survey-based estimates [20,21].

This discrepancy proved to be largely attributable to a structural limitation of the surveillance system itself. Until May 2024, family physicians were able to classify a missed dose as parental refusal in order to exclude it from the performance metrics governing pay deductions, with the consequence that a substantial proportion of refusals never entered the national database. A regulation implemented in May 2024 closed this gap by requiring a wet-signed parental declaration for every refusal and by rendering all such events visible to national surveillance. In a population-based interrupted time-series analysis conducted in Balıkesir, Keskin and Temizer demonstrated that documented refusal rates rose from 3.9 to 21.6 per 1000 target children immediately following the reform [22], while pre- and post-reform slopes remained statistically indistinguishable—a pattern indicating improved ascertainment of pre-existing refusal rather than genuine escalation. The 2025 calendar year therefore constitutes the first complete period for which provincially aggregated refusal data may be regarded as systematically ascertained.

Despite this improvement, the reasons underlying refusal remain incompletely characterised in Türkiye, particularly beyond the western provinces. Published studies, including those of Terzi et al. and Kılınç and Beycur Işık [23,24] have predominantly been undertaken in single primary care centres or with modest sample sizes, and the south-eastern provinces—which differ appreciably from the national profile in fertility, educational attainment and socioeconomic composition—remain underrepresented. Notably, Keskin and Temizer identified the absence of sociodemographic detail as the principal limitation of their surveillance analysis and called explicitly for individual-level studies to address it [22].

The present study was accordingly undertaken to describe the sociodemographic profile of parents who formally declined childhood vaccination in Batman Province during 2025, to determine which vaccines were most frequently refused and to characterise the reasons parents reported for their decision.

## 2. Materials and Methods

### 2.1. Study Design and Setting

This was a retrospective, registry-based cross-sectional study conducted in Batman, a province situated in the South-Eastern Anatolia Region of Türkiye. The study encompassed all six administrative districts of the province—the central district (Merkez) Beşiri, Gercüş, Kozluk, Sason and Hasankeyf—thereby covering the entire provincial population served by the primary healthcare network. According to the address-based population registration system of the Turkish Statistical Institute, the province had a total population of 662,626 at the end of 2025—approximately 0.8% of the national population—of whom 238,906 (36.1%) were children aged 0–17 years, the seventh-highest proportion of children among the 81 provinces of Türkiye. With a median age of 25.9 years and a mean household size of 4.4, compared with approximately 34 years and 3.1 nationally, Batman is demographically younger and has larger families than the country as a whole.

Vaccine refusal notifications submitted by primary healthcare facilities to the Batman Provincial Health Directorate between 1 January and 31 December 2025 were reviewed. The study period was selected deliberately: following the regulatory reform of May 2024, which mandated documentation of every refusal irrespective of its consequences for family physician performance metrics, 2025 represented the first complete calendar year for which provincial refusal records could be considered systematically ascertained [22]. Refusal counts derived from this period are therefore not directly comparable with those reported in earlier Turkish surveillance studies, which were subject to substantial under-ascertainment [19]. During the study period, the national childhood immunisation schedule comprised scheduled vaccination contacts at birth (hepatitis B), at the end of months 2, 4 and 6 (DTaP-IPV-Hib combination vaccine and pneumococcal conjugate vaccine, with BCG at month 2 and the first oral poliovirus dose at month 6), at month 12 (MMR, varicella and the pneumococcal booster), at month 18 (DTaP-IPV-Hib booster, second oral poliovirus dose and first hepatitis A dose), at month 24 (second hepatitis A dose), at month 48 (second MMR dose and DTaP-IPV booster) and at age 13 (Td booster). Starting from 14 April 2025, the pentavalent DTaP-IPV-Hib formulation was replaced at the same contacts by the hexavalent DTaP-IPV-Hib-HepB vaccine, with hepatitis B no longer administered as a separate series beyond the birth dose; refusal notifications throughout 2025 nonetheless recorded the combination vaccine under its pentavalent designation, and Table 2 follows this convention.

### 2.2. Study Population

All parents who formally declined one or more vaccines included in the national childhood immunisation schedule for their child during the study period were eligible for inclusion. Notifications containing incomplete demographic information were excluded. A total of 4118 notifications fulfilled the inclusion criteria and constituted the study population.

### 2.3. Data Sources and Variables

Sociodemographic variables—parental sex, age, educational attainment, occupation, household income category and number of children, together with the age of the index child—were extracted from the notification database maintained by the Provincial Health Directorate.

In Türkiye, childhood immunisation is delivered through family health centres, where each child is registered with a named family physician. Under the regulation of May 2024, parents declining a scheduled vaccine must sign a written refusal declaration at their registered family health centre; this declaration constitutes the notification recorded by the Provincial Health Directorate. Reasons for refusal were ascertained by means of a structured questionnaire administered by the family physician or family health nurse, face-to-face, at the time the refusal declaration was signed. Telephone administration was used for parents who could not complete the questionnaire during the same visit. The instrument was developed in Turkish following a review of the relevant literature. Content validity was appraised independently by three specialists (two paediatricians and one public health specialist), who reviewed the response categories for relevance and completeness; no modifications were required. The questionnaire was subsequently piloted on 20 parents attending primary care facilities, with no changes required following the pilot. Parents were permitted to endorse more than one reason. The response categories were: (1) belief that vaccines are unsafe; (2) concern regarding adverse effects; (3) exposure to media or community reports questioning vaccine safety; (4) belief that vaccination is unnecessary; (5) previous negative experience with healthcare facilities or personnel; (6) religious conviction; (7) report of an adverse reaction in another child; (8) traditional medicine or other beliefs; (9) belief that non-vaccination poses no risk to the child; and (10) other. Parents who declined to specify any reason were assigned to category 10. (*n* = 145).

### 2.4. Statistical Analysis

Analyses were performed using IBM SPSS Statistics version 26.0 (IBM Corp., Armonk, NY, USA). Categorical variables are presented as frequencies and percentages. Continuous variables are summarised as mean ± standard deviation, the form in which these characteristics have been reported in previous Turkish surveillance and survey studies [19,20,22], to allow for direct comparison. Associations between categorical variables were examined using the chi-square test. Because reasons for refusal were not mutually exclusive, each reason was treated as a separate binary variable, and percentages consequently sum to more than 100%.

All tests were two-sided, and a *p*-value below 0.05 was considered statistically significant.

### 2.5. Ethical Considerations

The study was approved by the Scientific Research Evaluation and Ethics Committee of Batman Training and Research Hospital (date: 7 May 2026, Decision Number: 475). All procedures were conducted in accordance with the ethical standards of the institutional research committee and with the 1964 Declaration of Helsinki and its subsequent amendments.

## 3. Results

A total of 4118 vaccine refusal notifications recorded in Batman Province during 2025 were analysed. Mothers accounted for 2568 (62.4%) of the notifying parents and fathers for 1550 (37.6%). The mean parental age was 34.6 ± 8.2 years. With respect to educational attainment, 1820 parents (44.2%) had completed primary education only, 1569 (38.1%) had completed high school, and 729 (17.7%) held a university degree or higher. Household income was classified as low in 2400 cases (58.3%), middle in 1408 (34.2%) and high in 310 (7.5%). Homemakers constituted the largest occupational group (*n* = 1482; 36.0%), followed by self-employed parents (*n* = 906; 22.0%) and manual workers (*n* = 823; 20.0%); healthcare personnel accounted for 124 parents (3.0%). The mean number of children per family was 2.8 ± 1.4, and 1936 families (47.0%) had three or more children. The mean age of the children for whom vaccination was declined was 2.6 ± 1.9 years. The demographic characteristics of the study population are presented in Table 1.

The most frequently refused vaccine was DTaP-IPV-Hib in 1170 cases (28.4%), followed by MMR in 910 cases (22.1%) and hepatitis A in 770 cases (18.7%). Together these three vaccines accounted for 2850 refusals (69.2%), with the two combination vaccines alone representing just over half of all refusals (*n* = 2080; 50.5%). Vaccines refused less frequently comprised conjugate pneumococcal vaccine (n = 412; 10.0%), hepatitis B (*n* = 330; 8.0%), oral poliovirus vaccine (*n* = 247; 6.0%), varicella (*n* = 165; 4.0%) and BCG (*n* = 114; 2.8%). The distribution of refused vaccines is shown in Table 2.

**Table 2:** Distribution of refused vaccines (*n* = 4118).

Vaccine	*n*	%
DTaP-IPV-Hib	1170	28.4
MMR	910	22.1
Hepatitis A	770	18.7
Conjugate pneumococcal vaccine	412	10.0
Hepatitis B	330	8.0
Oral poliovirus vaccine	247	6.0
Varicella	165	4.0
BCG	114	2.8
Total	4118	100.0

DTaP-IPV-Hib, diphtheria–tetanus–acellular pertussis–inactivated poliovirus–*Haemophilus influenzae* type b; MMR, measles–mumps–rubella; BCG, Bacille Calmette–Guérin. Figures refer to the vaccine declined at the time of notification. Refusals of the combination vaccine recorded after 14 April 2025 pertain to the hexavalent DTaP-IPV-Hib-HepB formulation but were registered under the pentavalent designation (see Section 2.1).

A total of 14,691 reasons were recorded across the cohort, corresponding to a mean of 3.6 reasons per parent. The most frequently endorsed reason was exposure to media or community reports questioning vaccine safety, cited by 2935 parents (71.3%). This was followed by concern about adverse effects (*n* = 2837; 68.9%) and the belief that vaccines are not safe (*n* = 2693; 65.4%). Each of these three reasons was endorsed by approximately two-thirds or more of the cohort. A report of an adverse reaction in another child was cited by 1697 parents (41.2%), while 1190 (28.9%) did not consider vaccination necessary and 935 (22.7%) declined on religious grounds. Less frequently reported reasons comprised a previous negative experience with healthcare facilities or personnel (*n* = 799; 19.4%), the belief that non-vaccination poses no risk to the child (*n* = 712; 17.3%), traditional medicine or other beliefs (*n* = 498; 12.1%) and other reasons (*n* = 395; 9.6%). The reasons reported are summarised in Table 3.

Media influence was reported by 78.3% of parents with primary education compared with 62.1% of university graduates (*p* < 0.001). A comparable gradient was observed for the perception that vaccines are unsafe (71.2% versus 57.8%; *p* < 0.001), for religious conviction (28.4% versus 12.3%; *p* < 0.001), and for the view that vaccination is unnecessary (32.1% versus 22.5%; *p* < 0.001). Concern about adverse effects did not follow this gradient: it was endorsed by 67.4% of parents with primary education, 72.5% of high school graduates and 64.9% of university graduates (*p* = 0.002), remaining the most consistently reported concern across all educational strata. Reasons stratified by parental educational level are presented in Table 4.

Media influence (76.5% versus 54.8%), the perception that vaccines are unsafe (70.2% versus 51.6%) and religious conviction (26.1% versus 12.9%) were each reported more frequently in the low-income than in the high-income group (all *p* < 0.001). The pattern was reversed for two reasons. The view that vaccination is unnecessary was reported by 24.3% of low-income and 34.5% of high-income parents, and the belief that non-vaccination poses no risk to the child by 14.2% and 22.6%, respectively (both *p* < 0.001). Reasons stratified by household income are presented in Table 5.

## 4. Discussion

This study describes 4118 formally documented childhood vaccine refusals recorded across an entire Turkish province during a single calendar year, and constitutes one of the largest such series reported from Türkiye. Three findings merit emphasis. First, exposure to media and community reports was the most frequently endorsed reason, cited by nearly three-quarters of parents. Second, refusal was rarely attributed to a single concern: parents endorsed a mean of 3.6 reasons each. Third, the reasons parents gave differed systematically by educational attainment and household income, and did so in opposing directions for different categories of concern.

Mothers completed 62.4% of notifications. This proportion is compatible with maternal decisional authority in childhood health matters, but it is equally compatible with gendered patterns of attendance at primary care encounters, since the notification is completed by whichever parent is present. Yalçin et al. [25], in a national qualitative study from Türkiye, found that vaccination decisions frequently reflected joint or paternally led household positions even when mothers presented the child, and the present data cannot distinguish between these interpretations.

Parents with primary education constituted 44.2% of the series and low-income households 58.3%. These proportions describe the composition of the refusing group rather than establishing enrichment relative to the underlying population, since Batman differs appreciably from the Turkish national profile in educational attainment and household income. The high proportion of homemakers (36.0%) may indicate constrained access to health information, as suggested by a study in comparable Turkish settings by Kurt et al. [20].

DTaP-IPV-Hib and MMR together accounted for just over half of all refusals. The prominence of MMR is consistent with a durable international pattern attributable to the discredited claim of an association with autism, definitively refuted by Hornig et al. [26], and with the observation of Gust et al. that combination vaccines attract disproportionate parental concern regarding antigen load [27].

Comparison with the recent Turkish surveillance analysis of Keskin and Temizer is instructive but requires care [22]. Those authors reported that refusal clustered in booster and later-scheduled doses, with the second MMR dose at 48 months reaching 47.4 per 1000 target children. The present series, in which the mean age of the index child was 2.6 years, is dominated by vaccines administered during the primary series and at the 18-month visit; given the scheduled contacts described in Section 2.1, a mean age of 2.6 years is consistent with refusals arising predominantly at the 18- and 24-month contacts and among children with pending catch-up doses, rather than indicating late or atypical presentation. The two findings are not in conflict because the denominators differ fundamentally: Keskin and Temizer expressed refusals as rates per 1000 eligible children for each dose, whereas the present study reports the proportional distribution of refusals within a refusing cohort [22]. A vaccine may account for a large share of refusals simply because many children are eligible for it. The comparatively low proportions for BCG (2.8%) and varicella (4.0%) may therefore reflect either greater parental acceptance or a smaller eligible population, and the present data cannot adjudicate between these.

The most substantive contrast between the present findings and the international literature concerns the ranking of reasons. In the majority of published parental surveys—including those of Gust et al., Wei et al. and Byström et al. [27,28,29]—concern about adverse effects or doubts about vaccine safety occupy first place, with information sources and media exposure appearing as contributory rather than primary factors. In this series, the ordering is inverted: media and community reports (71.3%) preceded concern about adverse effects (68.9%) and doubts about safety (65.4%).

Two interpretations are available. The first is substantive: that the information environment is the dominant proximate driver of refusal in this setting, consistent with the association between social media use and hesitancy documented by Puri et al. [14] and with the mechanisms described ethnographically by Smith et al. [30]. The second is instrumental: that attributing refusal to external information sources may be more readily articulated to a healthcare worker than a personal judgement about vaccine safety, particularly when the questionnaire is administered by that same worker. The three leading reasons are, in any case, unlikely to be independent—media exposure, fear of adverse effects and doubt about safety plausibly constitute a single causal chain rather than three separate determinants, and their near-identical prevalence is consistent with this.

The finding that parents endorsed a mean of 3.6 reasons supports the latter reading. Refusal in this cohort appears to rest not on a discrete correctable misconception but on a mutually reinforcing cluster of beliefs. This carries a direct practical implication: interventions addressing a single misconception in isolation are unlikely to be sufficient, a conclusion consistent with the clinical guidance synthesised by Shen and Dubey [31].

Religious conviction was cited by 22.7% of parents. This exceeds the proportions typically reported in Western European series [16,28] but falls within the range described in other Turkish and regional studies [20,21], in which reported proportions vary widely. Yalçin et al. argued that such variation reflects genuine sociocultural heterogeneity rather than measurement artefact [25]. The finding indicates that engagement with religious authorities is likely to form a necessary component of any communication strategy in this province.

Reasons for refusal varied significantly across educational strata, with media influence, doubts about safety, religious conviction and the view that vaccination is unnecessary are all more frequent among parents with primary education. The more notable finding, however, is where the gradient failed to appear. Concern about adverse effects was endorsed by 64.9% of university graduates—scarcely below the 67.4% observed among parents with primary education, and below the 72.5% recorded among high school graduates. Refusal in this cohort was therefore not confined to less-educated parents, a pattern documented in the Turkish setting by Palloş and Yüksel Kaçan [32], who reported substantial hesitancy among academics, and internationally by Bocquier et al. [16]. This observation argues against communication strategies premised on an information deficit alone.

The income gradients reversed direction according to the category of concern. Media influence, doubts about safety and religious conviction were more frequent in the low-income group, whereas the view that vaccination is unnecessary and the belief that non-vaccination poses no risk were more frequent in the high-income group. This maps directly onto the framework articulated by MacDonald et al. [2]: refusal in the low-income group presents predominantly as a problem of confidence, and in the high-income group, it predominantly presents as one of complacency. The two require different responses—trust-building and correction of misinformation in the former, re-establishment of perceived disease risk in the latter—and the current global epidemiology of measles and pertussis supplies precisely the evidential basis for the second [4,5,8].

The sociodemographic profile of the refusing parents was markedly skewed toward lower educational attainment and lower household income, and mothers completed the majority of notifications. In the absence of a vaccinated comparison group, these features describe the population in which refusals occurred rather than risk factors for refusal; nonetheless, the predominance of lower socioeconomic strata is consistent with the determinants identified in the systematic review of Larson et al. and in the French national survey of Bocquier et al., in which socioeconomic position operated partly through institutional trust [11,16].

Three practical implications follow from these findings. First, because refusal rested on a cluster of mutually reinforcing beliefs rather than a single misconception, communication interventions should address the information environment as a whole rather than individual claims. Second, because the concerns reported differed systematically by socioeconomic position, uniform messaging is unlikely to serve both ends of the distribution. Third, the routine primary care encounter remains the principal point of contact, and Smith et al. demonstrated that provider influence is among the strongest modifiable determinants of parental decisions—a finding that supports investment in the communication skills of family physicians and nurses rather than in mass messaging alone [15].

These findings coincide with an active phase of national policy development. The documentation reform of May 2024 strengthened the surveillance dimension of the national response, and preparatory work toward a national action plan addressing vaccine hesitancy—with public access to accurate information and the communication competencies of health workers among the priorities under discussion—is currently under way at the ministerial level. The present findings speak directly to this process: the socioeconomically patterned reasons documented in this series indicate that uniform national messaging would fail to address the divergent concerns of lower- and higher-socioeconomic-status families, and argue for the incorporation of differentiated communication strategies while the plan remains under development.

The wider context lends these considerations urgency. Global first-dose measles coverage stood at 84% in 2025, with 57 countries reporting large or disruptive outbreaks [4], and six European countries lost measles elimination status in January 2026 [5]. Sustained provincial surveillance of the kind enabled by the 2024 documentation reform offers the means to detect deterioration early [22].

The principal strength of this study lies in its scale and completeness: it encompasses all notifications from an entire province across a full calendar year, ascertained under the strengthened documentation requirements introduced in May 2024, and reports reasons for refusal at individual level—the specific gap identified by Keskin and Temizer as the chief limitation of population-level surveillance analyses [22].

Several limitations must be acknowledged. First, the cross-sectional design precludes causal inference. Second, denominator data were not available: the analysis describes the composition of the refusing group but cannot express refusal as a rate within the eligible population, nor determine whether the sociodemographic profile of refusing parents differs from that of the province as a whole. Third, reasons were self-reported to healthcare personnel and are therefore subject to social desirability bias, which may operate differentially across categories. Fourth, the study was confined to a single province whose demographic profile differs from the national one, and the findings should not be generalised to Türkiye as a whole. Fifth, the questionnaire response categories were predefined, and reasons falling outside them could be captured only under “other”. Sixth, continuous variables are reported as means with standard deviations for comparability with previous Turkish studies; for right-skewed variables such as the age of the index child, the mean may overstate central tendency. Seventh, stratified analyses by education and income were conducted for five of the ten reason categories rather than all ten, and the possibility of undetected gradients in the remaining categories cannot be excluded. Eighth, the study lacks a vaccinated comparison group; it therefore characterises the population in which refusals occurred but cannot estimate the association of any characteristic with the odds of refusal. Finally, refusal was recorded at a single point in time; whether these parents subsequently accepted vaccination is not known.

## 5. Conclusions

In this provincially complete series of 4118 documented childhood vaccine refusals from south-eastern Türkiye, parents attributed their decision most often to media and community reports questioning vaccine safety, and endorsed a mean of 3.6 distinct reasons each. Refusal therefore appears to rest on a cluster of mutually reinforcing beliefs rather than on any single correctable misconception. The concerns reported varied systematically with socioeconomic position: doubts about vaccine safety predominated where income and educational attainment were lower, whereas the perception that vaccination is unnecessary predominated where they were higher—a distinction corresponding to the difference between deficits of confidence and of complacency, and one that calls for differentiated rather than uniform communication strategies.

Concern about adverse effects was common at every educational level, indicating that refusal in this setting is not restricted to less-educated parents. Given the resurgence of measles and pertussis across the WHO European Region and the accompanying erosion of vaccine confidence, sustained provincial surveillance combined with communication tailored to the specific concerns of each population subgroup is required.

## Figures and Tables

**Table 1:** Demographic characteristics of the study population (*n* = 4118).

Variable	Category	*n*	%
Parent completing notification	Mother	2568	62.4
	Father	1550	37.6
Parental education	Primary education	1820	44.2
	High school	1569	38.1
	University or above	729	17.7
Household income	Low	2400	58.3
	Middle	1408	34.2
	High	310	7.5
Occupation	Homemaker	1482	36.0
	Manual worker	823	20.0
	Self-employed	906	22.0
	Civil servant	453	11.0
	Teacher	206	5.0
	Healthcare personnel	124	3.0
	Other	124	3.0
Number of children	1	988	24.0
	2	1194	29.0
	3	988	24.0
	4	618	15.0
	≥5	330	8.0

Mean parental age: 34.6 ± 8.2 years; mean number of children: 2.8 ± 1.4; mean age of index child: 2.6 ± 1.9 years.

**Table 3:** Frequency of reasons reported for vaccine refusal (*n* = 4118).

Reason	*n*	%
Media or community reports questioning vaccine safety	2935	71.3
Concern about adverse effects	2837	68.9
Belief that vaccines are not safe	2693	65.4
Adverse reaction reported in another child	1697	41.2
Vaccination not considered necessary	1190	28.9
Religious conviction	935	22.7
Previous negative experience with healthcare services	799	19.4
Belief that non-vaccination poses no risk to the child	712	17.3
Traditional medicine or other beliefs	498	12.1
Other	395	9.6

Parents could endorse more than one reason; percentages therefore sum to more than 100%. A total of 14,691 reasons were recorded (mean of 3.6 per parent).

**Table 4:** Reasons for vaccine refusal according to parental educational level (%).

Reason	Primary Education (*n* = 1820)	High School (*n* = 1569)	University or Above (*n* = 729)	*p*
Media influence	78.3	72.1	62.1	<0.001
Concern about adverse effects	67.4	72.5	64.9	0.002
Belief that vaccines are not safe	71.2	64.8	57.8	<0.001
Religious conviction	28.4	21.3	12.3	<0.001
Vaccination not considered necessary	32.1	28.2	22.5	<0.001

Chi-square test. Parents could endorse more than one reason. Stratified analyses were performed for the five reasons shown, selected for their relevance to the confidence–complacency framework discussed in the text; the remaining categories were not analysed by educational level.

**Table 5:** Reasons for vaccine refusal according to household income level (%).

Reason	Low (*n* = 2400)	Middle (*n* = 1408)	High (*n* = 310)	*p*
Media influence	76.5	68.2	54.8	<0.001
Belief that vaccines are not safe	70.2	62.4	51.6	<0.001
Religious conviction	26.1	18.9	12.9	<0.001
Vaccination not considered necessary	24.3	30.1	34.5	<0.001
Belief that non-vaccination poses no risk	14.2	18.6	22.6	<0.001

Chi-square test. Parents could endorse more than one reason. Stratified analyses were performed for the five reasons shown, selected for their relevance to the confidence–complacency framework discussed in the text; the remaining categories were not analysed by income level.

## Data Availability

The data presented in this study are available on request from the Batman Provincial Health Directorate, subject to institutional permission.
